# Implementation of High-Throughput Sequencing (HTS) in Aptamer Selection Technology

**DOI:** 10.3390/ijms21228774

**Published:** 2020-11-20

**Authors:** Natalia Komarova, Daria Barkova, Alexander Kuznetsov

**Affiliations:** Scientific-Manufacturing Complex Technological Centre, 1–7 Shokin Square, Zelenograd, 124498 Moscow, Russia; daria.barkhova@gmail.com (D.B.); kae@tcen.ru (A.K.)

**Keywords:** aptamer, SELEX, high-throughput sequencing, next-generation sequencing, random library, evolution

## Abstract

Aptamers are nucleic acid ligands that bind specifically to a target of interest. Aptamers have gained in popularity due to their high potential for different applications in analysis, diagnostics, and therapeutics. The procedure called systematic evolution of ligands by exponential enrichment (SELEX) is used for aptamer isolation from large nucleic acid combinatorial libraries. The huge number of unique sequences implemented in the in vitro evolution in the SELEX process imposes the necessity of performing extensive sequencing of the selected nucleic acid pools. High-throughput sequencing (HTS) meets this demand of SELEX. Analysis of the data obtained from sequencing of the libraries produced during and after aptamer isolation provides an informative basis for precise aptamer identification and for examining the structure and function of nucleic acid ligands. This review discusses the technical aspects and the potential of the integration of HTS with SELEX.

## 1. Introduction

Aptamers, short single-stranded DNA or RNA molecules capable of specific binding to a target of interest, are gaining in research interest due to their versatile application potential as analytical, diagnostic, and therapeutics agents. Aptamers offer the advantages of low synthesis cost, small size, thermal stability, ease of labeling, and ability of regeneration. aptamers are usually obtained using the systematic evolution of ligands by exponential enrichment (SELEX) process, which was first implemented in the 1990s [1,2]. During its three decades in existence, SELEX has obtained modifications aiming to extend the target range and improve the efficiency and speed of the selection process.

SELEX is an iterative procedure of selection of target-binding species from a vast library of random oligonucleotides. The random nucleic acid library is incubated with the target molecules to facilitate the formation of a complex between the target and oligonucleotides displaying target affinity. Target-bound species are then separated from the rest of the unbound library pool and PCR-amplified. The new ssDNA/RNA library enriched with target-binding oligonucleotides is then regenerated from the PCR product and involved in the novel selection cycle. The selection continues until the library is sufficiently enriched.

The progress of enrichment can be monitored with the simple detection of the amount of target-bound nucleic acid by measuring the affinity of the selected oligonucleotide pool to the target or by assessing the decrease in the diversity of the selected library. In conventional SELEX, the terminal library after the selection is cloned and 30–100 representatives are sequenced with Sanger sequencing. Few of them are picked either randomly or based on sequence analysis for further experimental evaluation of their affinity and specificity to the target, after which the candidate oligonucleotide can be nominated as the aptamer. In that respect, the correct identification of candidate aptamers is a key point for the overall selection success [3].

The huge progress in DNA sequencing technologies, enabling high-throughput sequencing (HTS) of DNA and RNA molecules of different lengths, has occurred within the past fifteen years with the establishment of next-generation sequencing technologies that found an application in SELEX technology. The starting library for aptamer selection normally contains 10^11^–10^16^ unique nucleic acid species; the library diversity is practically never sufficiently reduced with enrichment under SELEX to a size that can be effectively covered with Sanger sequencing. Therefore, the ability to identify up to millions of sequences provided by HTS instead of hundreds with classic sequencing perfectly fits the SELEX procedure.

The enlarged number of recognized SELEX-derived oligonucleotide sequences enables more precise candidate aptamer identification. In addition, HTS applied for cycle-to-cycle enriched libraries can elucidate the constraints and pitfalls of the aptamer isolation process, which in turn can provide the basis for rendering SELEX more efficiently. The main drawback of HTS is the generation of an enormous amount of data to be analyzed. The potential of HTS data is often not realized due to the complexity of data processing, the need for high expertise in bioinformatics, and the lack of easy-to-use software [4]. This review discusses the practical implementation of HTS to SELEX technology with an emphasis on the potential of HTS-SELEX to provide more information on the SELEX process and aptamer structure–function relationship reached by analyzing HTS data.

## 2. Sequencing Platforms

Next generation sequencing techniques include several different strategies that allow short or long reads of the target nucleic acid molecule [5]. High-throughput sequencing in SELEX is mainly shared between the Illumina, 454 Roche, and IonTorrent sequencing platforms. The Illumina sequencing platform is believed to be the most widespread [6]. All of these platforms employ sequencing-by-synthesis (SBS) techniques and enable short reads of the target nucleic acids. The read length is limited to 300–400 nt depending on the specific device. Commonly, the starting SELEX library does not exceed 150 nt [3], and the length of short-read sequencing is sufficient to read the entire sequence of library representatives.

**Figure 1:**
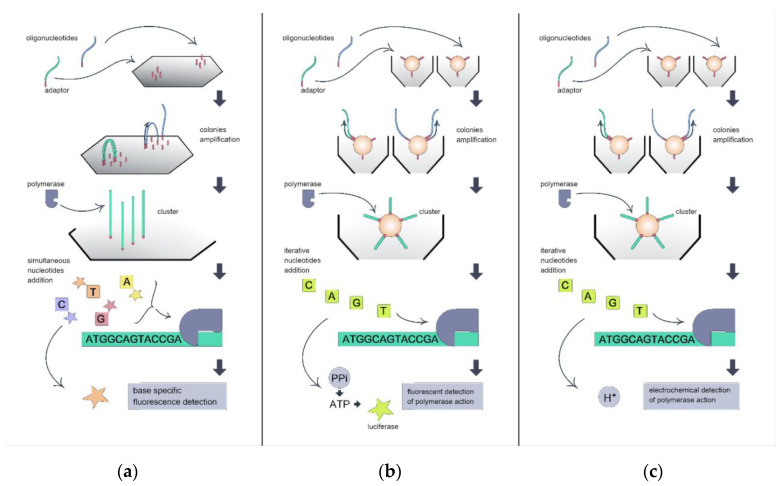
Illustration of sequencing-by-synthesis technology variants implied by different high-throughput sequencing platforms: (**a**) Illumina; (**b**) 454 Roche; and (**c**) IonTorrent.

In SBS technology, the sequenced DNA strand serves as the template for the synthesis of the complementary strand, and the sequencing device identifies the bases incorporated into this synthesized strand. Before sequencing, the target DNA is clonally amplified to achieve localization of the same single-stranded sequence clones at the distinct area of the sequencing chip. This cloning serves for signal amplification during the sequencing process (Figure 1). In Illumina technology (Figure 1a), the clones of each single template molecule are amplified directly above the sequencing chip surface using bridge amplification [5]. As a result, the clones of each initial template sequence form a cluster of a detectable size on the chip.

The colonies of the ssDNA template are then hybridized with the sequencing primer. Then, the DNA polymerase begins the synthesis of the complementary strand. Four fluorescently modified dNTPs are simultaneously added to the chip surface for each synthesis step. At each colony cluster, the base-specific fluorescent signal is formed with base incorporation, and this signal is recognized by the device detector. In 454 Roche (Figure 1b) and IonTorrent (Figure 1c) devices, clones of a single template molecule are formed on the surface of the beads using the emulsion PCR process. A single bead, a single template molecule, DNA polymerase, dNTPs, and primers are captured in the emulsion drop, and, after amplification, the bead is covered with the clones of the template.

Then, the beads are distributed above the cell-structured sequencing chips, enabling further signal detection. Four different dNTPs are iteratively added to the chip. If the added dNTP is complementary to the template DNA, the dNTP incorporates the synthesized strand, and DNA polymerase action generates the signal to be detected by the device. In 454 Roche devices, the release of pyrophosphate during each DNA polymerization step is fluorescently detected with the use of an enzyme cascade. IonTorrent devices electrochemically detect the release of H^+^ at each DNA polymerization step using an ion-sensitive field effect transistor (ISFET).

Next generation sequencing (NGS) techniques enabling long reads such as Nanopore sequencing are less applicable for sequencing SELEX libraries. Theoretically, long reads can be useful in genomic RNA SELEX, where the initial library species can be longer than in conventional SELEX [7]. The ability of nanopore sequencing for the detection of modified nucleobases can also be applicable in the process of isolating aptamers containing modified and unnatural bases [6].

## 3. Sample Preparation for High-Throughput Sequencing (HTS)

HTS always requires special library preparation before sequencing. In SBS, library preparation is intended to enable the correct clonal amplification of the substrate and to introduce the universal primer binding site for further enzymatic synthesis of the complementary strand for signal generation. For this purpose, the ssDNA/RNA pool obtained from SELEX is transformed to dsDNA flanked with special adaptor sequences. These adaptors contain specific sequences that serve both the hybridization of the target DNA with the complementary short ssDNA strands on the surface of the solid support for further clonal amplification and in the priming of DNA synthesis. In addition to the regions necessary for hybridization and amplification of the template, the adaptor sequences can bear specific indices or barcodes to encode the sample, which allows for the sequencing of multiple samples within a single chip. This option is especially relevant for the high-throughput sequencing of the SELEX libraries obtained from cycle to cycle under the selection process.

**Figure 2:**
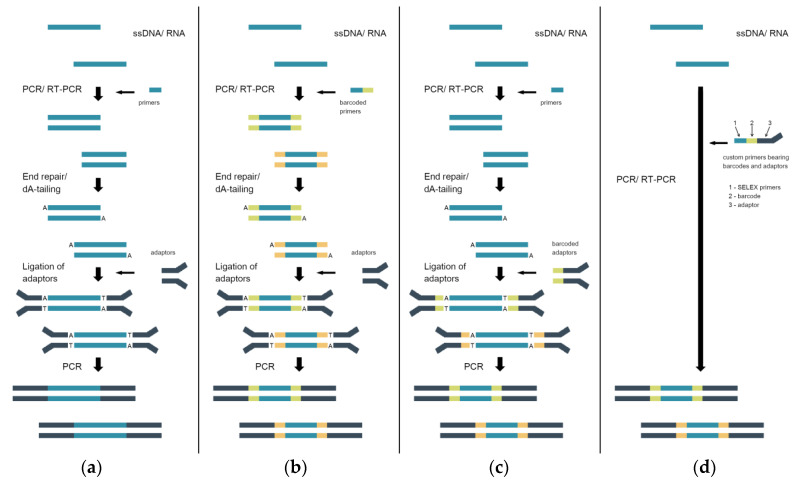
Sample preparation workflows for sequencing library preparation. (**a**) The common supplier’s workflow for the preparation of non-indexed libraries. (**b**) The workflow for the preparation of indexed libraries; barcodes are introduced at the first PCR amplification step with the custom primers. (**c**) The workflow for the preparation of indexed libraries; barcodes are introduced at the ligation step using the commercial adaptors. (**d**) Single PCR workflow for the preparation of indexed libraries; barcodes and adaptors are introduced with the custom primers.

Sequencing adaptors and kits for their incorporation can be purchased from commercial suppliers. The sample preparation workflow for different SBS platforms is similar and includes the following steps: the conversion of ssDNA or RNA to dsDNA, end repair (blunt-ending and the phosphorylation of 5′-ends), adenylation of 3′-ends, ligation of adapters, selection of correct ligation product, and amplification of the adaptor-ligated product (Figure 2a). The last PCR-amplification step is optional, and should be avoided for SELEX libraries to escape additional PCR bias [8,9]. This also shortens the sample preparation time.

The only requirement is the enlarged amount of the starting dsDNA sample, which is affordable in most SELEX experiments. The majority of sample preparation kits are developed for the preparation of sequencing libraries for large DNA and RNA molecules and, therefore, assume mechanical or enzymatic fragmentation of the sample. For SELEX-derived libraries, this step is not needed [10]. The majority of protocols for commercial kits also employ the usage of magnetic AMPure XP beads for the size-selection of DNA fragments after the adaptor ligation step. SELEX-derived samples are typically too small for bead-based separations and, therefore could be better (but not obligatory [11]) processed using gel-based separations and extraction [10]. Both agarose and polyacrylamide gels are acceptable.

As an example, a detailed sample preparation protocol for Illumina sequencing based on a commercial PCR-free adapter ligation reagent kit is provided in [10]. Interestingly, it was suggested that sample specific barcodes be introduced in the first step of ssDNA PCR conversion to dsDNA with indexed primers instead of using indexed commercial adaptors (Figure 2b). The same sample barcoding approach was used in [12]. This makes sample preparation less expensive if sample pooling is used as it enables using only the adapter sequence for the final library, and this is especially relevant when ordering a commercial sequencing service [12]. Alternatively, barcode-containing adaptors can be used if necessary (Figure 2c).

The presence of fixed flanking regions in SELEX libraries allows one to omit the ligation of commercial adapters. For example, in [13], the samples obtained from different selection cycles were PCR-amplified with custom primers bearing priming sequences specific for the SELEX library, barcode sequences for encoding the samples from different selection cycles, and Illumina adapter sequences P5/P7 (Figure 2d). The sequencing sample was generated using only one PCR-amplification step with no need for subsequent adaptor ligation and additional amplification. The same approach employing fusion primers can be applied for other sequencing platforms [11,14,15].

A special library preparation protocol for Illumina sequencing is described for genetic alphabet expansion SELEX (ExSELEX), which employs an unnatural base pair system for the initial SELEX library [16]. SBS is normally unavailable for the libraries containing modified and unnatural nucleobases. To make the library compatible with deep sequencing, a special protocol was developed [16]. The initial SELEX library is designed to contain a few unnatural bases in each representative sample. Each unnatural base position is concatenated with a specific sequence of natural bases. After the selection process, the enriched library proceeds with two-step amplification in which unnatural bases are substituted with natural ones and, thus, becomes SBS-compatible. After sequencing, the known specific sequences serve as a kind of a barcode to recognize the positions of the unnatural base.

The sequencing libraries require thorough validation before sequencing because the efficiency of clonal amplification, and thus the sequencing output, depends strongly on the library concentration. Both the library length confirming the correct insertion of the initial template between the adaptor sequences and the library concentration should be checked. The library size can be confirmed using standard gel separation or the Bioanalyzer system. The best method for the determination of the library concentration is real-time PCR with special kits for the validation of NGS libraries as this specifically detects the adaptor-containing sequences that are essential for clonal amplification [10]. However, if the library is clean enough and displays the correct size with no by-products, the quantification can be done with sensitive fluorimeters like Qubit [10]. If the multiplexed library is sequenced, the concentration of different samples should be normalized before sample pooling to achieve equal (or desired) reads distribution between the sequences.

## 4. Processing of HTS Data

The use of HTS-SELEX techniques for aptamer selection requires new bioinformatics approaches to analyze the huge amount of information produced by NGS that would make the identification of aptamer candidates based on the sequencing results more transparent and reasonable. Simultaneously with the development of software for direct HTS-SELEX data analysis, software solutions appeared for practical research issues related to HTS-SELEX, for instance, reducing SELEX cycles [17,18,19,20,21], determining selection conditions [22], or even searching for binding sites [23,24]. The standard protocol for the analysis of HTS results can be divided into several stages: data preprocessing, primary enrichment analysis, library clustering, and a binding motifs search. Separate bioinformatics instruments to perform each of these steps individually exist, and many of them have been further integrated into more complex software packages to perform full-scale analysis of HTS data.

Data preprocessing steps depend on the structure of the library and the sequencing method [18]; however, in general, preprocessing consists of the removal of sequencing artifacts, filtering by sequence length, replacement of reverse sequences, and, in some cases, removal or masking regions of constant sequence. Initially, each laboratory solved these problems individually using in-house developed software [19,25], and also adopted Tallymer [26] software initially developed for k-mer sequence counting by setting the k-value to the length of the library random region [17]. Researchers with advanced computer competences can easily perform these tasks, but special programs and adaptations of existing platforms also appear to manage HTS-SELEX preprocessing. However, data preprocessing scripts are still aimed toward the classic SELEX design and are not adapted to other variations. For example, many programs do not allow for the masking of constant regions that are not at the edges of sequences, much like the Capture region in oligonucleotides when a selection has been performed according to the Capture-SELEX protocol [27].

The Galaxy Project is a convenient solution for researchers without bioinformatics skills. The platform is easy to use and contains a large number of standard text and bioinformatics tools that allow all steps of raw NGS data preprocessing to be performed [28]. W. H. Thiel compiled a workflow containing all the necessary stages of basic data preprocessing and the first stages of analysis to eliminate unenriched sequences [29] so that the initial pool of sequences could be reduced by more than 90%, facilitating and accelerating subsequent analysis. Another special program developed for the preprocessing of HTS-SELEX data is AptaPLEX [30], which is described further as it was included in a large program package for full HTS-SELEX data analysis.

Primary estimation of the enrichment of SELEX libraries is based on counting the copy number of each sequence in different rounds and identifying the number of rounds in which the sequence is present. These data can serve for the evaluation of the enrichment rate of each unique sequence throughout the library evolution under the selection pressure [19]. The Galaxy server offers tools for creating a non-redundant sequences database, which can further be exported and used for further analysis of the sequence abundance scoring and their cycle-to-cycle enrichment rate using any other desired instrument. These parameters, which are also calculated by most other programs, are used to track the dynamics of selection and are the basis for the identification of aptamer candidates. However, an assumption that the most enriched sequences take the lead in selection due to their ability to bind to the target is not always confirmed empirically, particularly with the increase in selection cycles [19,25,31]. Therefore, different programs perform sequence clusterization or binding motifs in the search for a more representative aptamer identification.

Clusterization aims to find sequence families and estimate the enrichment of each cluster instead of the enrichment of individual sequences. Clusterization, according to the primary structure, is based on the assumption that a random selection of oligonucleotides with near-identical primary structure from an underrepresented library is extremely unlikely; a supposal that this similarity is caused by some divergent biases during library processing during SELEX is more expectable. This clustering approach provides an opportunity to identify primary and mutant oligonucleotide forms, reduce the diversity of an analyzing pool, and select oligonucleotides with different structures for further screening [32]. The most common representatives of obtained clusters are likely to be different in structure and could interact with the target in different ways during selection [32].

FASTAptamer [32] is perhaps one of the most common software products for HTS-SELEX data analysis, especially in the primary stages. The program has the form of an open source script set, where each script aims at solving different problems and is usable regardless of the specifics of the SELEX experiment. The program has no graphical interface and, therefore requires at least the simplest understanding of working with the command line. FASTAptamer can be used on any operating system if the PERL interpreter is preinstalled that the program is written on. At the same time, FASTAptamer scripts are available on the previously described Galaxy platform (if it is installed on the computer) and can be combined into one workflow with data preprocessing.

The obligatory first stage of the analysis is the FASTAptamer-Count script, which uses the initially preprocessed sequencing data to calculate and normalize the frequency of unique sequences occurring in the pool (e.g., to perform primary estimation of the enrichment). The results of the FASTAptamer-Count are used by other scripts for sequence clustering, comparing the enrichment in different populations, and identifying the sequences with the highest enrichment rate over several rounds of selection. The clustering script is based on the Levenshtein distance method, which can compare sequences of different lengths [32]. PATTERNITY-seq, another clustering program, works on a similar principle, but has apparently not become widespread [33].

Another approach to cluster sequences based on their primary structure is the local hashing algorithm. Programs based on this algorithm work faster with a large amount of data; however, they are not capable of comparing sequences of different lengths, and therefore all sequences must be filtered to the same length before analysis [34]. This approach has been implemented in the programs AptaCluster [35] and SEWAL [36]. AptaCluster is described further as AptaSUITE. SEWAL was adapted for the analysis of Illumina results and provides analysis results in the form of three-dimensional scatter plots describing the accumulation of the sequences and their belonging to a specific cluster. The program does not have a graphical interface, and, if the user is using a non-Apple Macintosh operating system, the source code must be compiled before installation.

The significantly reduced pool, sized to tens or hundreds of sequences, can be subjected to the next stage of analysis based on the primary structure: the search for text motifs. For these purposes, the programs of the MEME Suite project [37] adapted for motif searching and analysis (MEME or GLAM) are the most often used. However, the programs do not predict the secondary structure of aptamers. The search for text motifs of various lengths among all sequences without prior data reduction processing can also be performed with the program MPBind [24], which evaluates the relative change in frequency and accumulation to the last rounds for all n-mer motifs. However, the program is written for Unix operating systems and requires advanced computer skills.

Folding structural approaches provide more complete information regarding the possible interaction of oligonucleotides with the target. These were applied earlier, when single sequences were randomly selected from the entire pool that successfully bound to the target [38]. However, in the context of HTS-SELEX analysis, a large-scale search for folding motifs is particularly relevant. In the course of library enrichment, the same folding structure can successfully accumulate; however, if it consists of fragments with different sizes or locations in a primary structure, text aligners would ignore this structure. However, most of the published folding searching approaches possess significant limitations.

Typically, they identify supposedly single-stranded regions (for instance AptaTRACE, which is a part of AptaSUITE described below) equating to a target binding site, and do not recognize other variants that may also be critical for complex formations. A successful motif search depends on the fine tuning of the parameters as biologically active folding is not always the most energetically favorable structure and can be stabilized by its target. In addition, such programs are focused on RNA libraries that can also distort the analysis if the library consists of oligonucleotides with other structures. The constant sequence regions should not be deleted or masked for the correct usage of a folding searching program as they can contribute to the formation of the secondary structure [39].

One of the first programs to search for aptamer folding motifs was Aptamotif [40]. The analysis is carried out in three stages: first, the optimal and suboptimal secondary structures are calculated using the RNAsubopt algorithm [41], then, the discovered structural fragments are extracted, successfully aligned fragments are selected for the next stage, and the most efficient variants are used to analyze the entire sequence library. The user needs to select the energy range of possible conformations to maintain a balance between the sensitivity and computational complexity. Another limitation is that the pool must be sufficiently enriched in binding aptamers—from 50% or more—since the algorithm does not equalize the entire pool, but only some of its random samples. Therefore, this approach may not give positive results for selection experiments with a high background binding level or should be used for datasets where non-specific sequences have been excluded.

APTANI was developed on the basis of Aptamotif [23]. In addition to analyzing secondary structures, it realizes some other functions such as aptamer clusterization by comparing the results of different HTS-SELEX cycles and searching for specific binding motifs. The updated version of the program, APTANI^2^ [42], has a graphical interface and provides more complete information regarding the enriched aptamers; moreover, it is capable of identifying a new variety of secondary structures including a rather important folding into G-quadruplex.

Other programs conduct comprehensive analysis of HTS-SELEX data using several different approaches to identify aptamers. For example, AptCompare [43] is a combination of AptaCluster [35], FASTAptamer [32], MPBind [24], APTANI [23], RNAmotifAnalysis [44], and a number of other scripts for preprocessing.

The proprietary software COMPAS performs complex data processing [45] and conducts analysis and clustering with different approaches that can be applied: comparison of full-length sequences or their 4–8 nucleotide k-mers, and comparison by the criteria of Shannon information entropy. The families grouped on the basis of the primary structure are combined with each other to form superfamilies according to possible similarities in the secondary structure. The program checks the input data and partially preprocesses them.

RaptRanker [46] also carries out complex data analysis. In the first stage, it passes all sequences through a rough filter, however, they must exactly match in length and in the sequence of constant regions. For unique sequences that have passed the preprocessing filter, a sequence structural profile is determined using the CapR algorithms [47], and clustering is performed according to a sub-sequence structural profile. The selection of specific candidates is based on the average motif enrichment that depends on the frequency of each subsequence, motif frequency, and motif enrichment for each cluster.

Finally, AptaSUITE [48] is a multifunctional program and a combination of previously published programs that carry out various stages of the analysis of HTS-SELEX data such as the previously mentioned AptaPLEX, AptaCLUSTER, and AptaTRACE as well as a program identifying polymerase errors AptaMUT [22] and simulator of HTS-SELEX—AptaSIM [48]. The AptaSIM program can be used for both adjusting the SELEX parameters and for an estimation of the influence of various factors on a real selection.

The program AptaPLEX [30] is used for data preprocessing. It sorts the obtained sequences according to barcodes into separate cycles, identifies and removes primers, and automatically corrects mismatches between forward and reverse reading. The combination of the programs analyzing the primary structure AptaCLUSTER and AptaMUT describes the dynamics of the pool enrichment, the representation of various clusters during the selection, and the accumulation of mutations in the clusters and their possible effect on the ability to bind to the target. The folding analysis is carried out by the AptaTRACE program, which indicates structures such as hairpins, bulge loops, inner loops, multiple loops, and dangling ends and evaluates their enrichment during selection. AptaSUITE is a cross-platform program and has both GUI and command line interfaces that make it convenient for people with different IT competencies.

In summary, the following different strategies can be outlined for the recognition of candidate aptamer sequences. The first is the abundance criteria. This approach arises from the initial basis of the SELEX experiment, which presumes the accumulation and enrichment of target specific sequences. The estimation of the enrichment rate from one selection cycle to another is a second approach for the discovery of aptamer sequences. This strategy not only accounts for the abundance, but also for the persistence of the individual sequence throughout the evolution process, and can help distinguish between the target-induced enrichment and biased sequence accumulation due to other non-specific reasons.

The third strategy relies on clusterization of the sequence dataset. In this case, prominent candidates are selected from different sequence clusters. As a result, less abundant but effective binders can be identified, while the sequences with the highest abundance and enrichment rate scores can originate from similar sequences with a moderate binding ability, which appear to be overrepresented due to stochastic reasons or selection bias. Finally, one further strategy for aptamer identification originates from the notion that not the entire oligonucleotide sequence but rather a rather short region exerts target binding; based on this, the enrichment of binding motifs instead of long sequences should serve for the recognition of aptamer sequences.

This strategy requires, first, the discovery of overrepresented binding motifs, which may both be structural sequences. The extracted motifs can be further clustered to achieve more precise aptamer identification. No systematic comparison of different strategies and software solutions for candidate aptamer identification is available at the moment; however, in certain cases, research articles devoted to the development of particular bioinformatics tools describe a comparison to other similar instruments [23,24,46].

Any of the described strategies or a combination of them can be applied in practical SELEX experiments. So far, the choice of software tools for the analysis of HTS experimental results depends primarily on the desired aptamer identification strategy. The computer competence of the researcher and the available user operating system can influence the software choice. Abundance scoring can be executed using almost all described software packages.

The cycle-to-cycle enrichment rate can be assessed using Galaxy, FASTAptamer, AptaSUITE (AptaPLEX), and RaptRankler. Cluster analysis can performed using ASTAptamer, AptaSUITE (AptaCluster), and SEWAL. A clusterization function is also available with Galaxy, but it is not adapted for SELEX experiments. Sequence motifs can be extracted using FASTAptamer, AptaSUITE, MPBind, MEME/GLAM (available with Galaxy or separately), and Tallymer. Structural motifs can be identified using APTANI, APTANI^2^, AptaSUITE (AptaTRACE), and RaptRanker. APTANI, MPBind, and RaptRanker are intended to provide rankings of aptamer candidates with prospective binding ability, thus, practically allows the user to escape rendering a final decision on candidate selection.

In relation to computer skills requirements, Galaxy is an absolutely outstanding tool due to its simplicity and availability for all users. The FASTAptamer and AptaSUITE packages require minimal user experience for the installation and command line operation. The execution of APTANI, APTANI^2^, RaptRanker, SEWAL, or MPBind requires more complex installation. APTANI and APTANI^2^ also demand some bioinformatics experience of the researcher due to the need to apply user-defined parameters. The functionality and characteristic features of the different HTS-SELEX software tools are summarized in Table 1.

## 5. Insights to Aptamer Selection Process Derived from HTS Data

**Figure 3:**
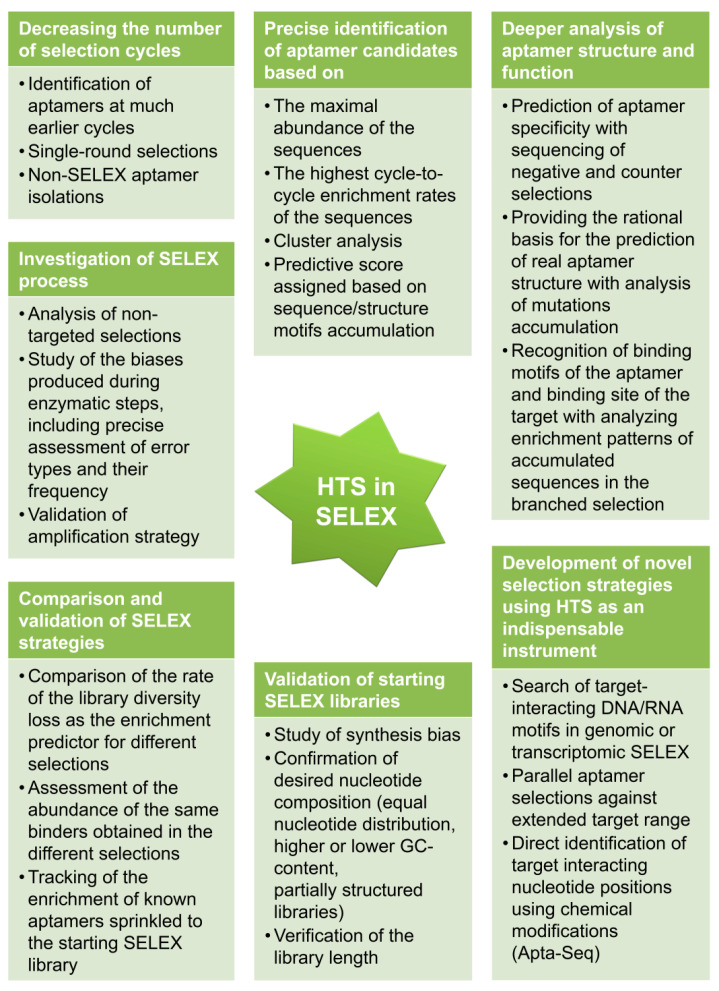
Outlines of the progress in SELEX obtained with HTS implementation.

Launching a HTS experiment with the SELEX process resulted in significant progress for aspects related to aptamer selection. These points are discussed below and summarized in brief in Figure 3.

### 5.1. Identification of the Most Prominent Candidate Aptamer Sequences

The main goal of introducing HTS to SELEX is the enlargement of sequenced species to enable a more precise selection of aptamer candidates, and all the bioinformatics methodology described above is intended to fit this goal.

First, the benefits of HTS implementation for candidate aptamer identification compared to classic Sanger sequencing was demonstrated experimentally. Increasing the number of sequenced species from 88 in Sanger-assisted FluMag-SELEX against Protein A (a structural part of the cell wall of the bacterial pathogen *S. aureus*) to 2597 resulted in the identification of two more effective aptamers [49]. The best aptamer could be recognized using both Sanger sequencing and HTS; however, from the HTS dataset, the potential was clearer. Notably, in this experiment, end-point sequencing of the terminal SELEX library was performed, and the obtained sequence dataset was not too large, thus, requiring less extensive and complex bioinformatics efforts, but still provided benefits in terms of candidate aptamer identification.

The use of more complex HTS of cycle-to-cycle enriched SELEX libraries in comparison to end-point Sanger sequencing demonstrated even more efficient candidate aptamer identification [50]. The aptamers against breast cancer cells were not at all recognized by Sanger sequencing; however, the HTS results revealed the enrichment of target binders at much earlier rounds [50]. Many other research works have demonstrated the ability of HTS for early aptamer identification [31,51,52]. An interesting comparison of Sanger and the HTS SELEX results was performed under selection against the Murine IL-10 Receptor [52]. The library obtained at the fifth selection round was subjected to HTS, and successful aptamers were identified with HTS data analysis. Then, the selection was continued, and pools from cycles 6, 9, 12, and 16 were Sanger sequenced.

The aptamers derived from cycles 6 and 9 exhibited low target binding. The most abundant sequence from the twelfth selection cycle was identical to the best aptamer identified with HTS at cycle 5. After 16 selection cycles, this binder had lost its abundance and was displaced with another aptamer with moderate target affinity. Some of the potent aptamers identified with HTS were not found using Sanger sequencing at all [52]. In the selection of aptamers against the platelet derived growth factor BB (PDGF-BB) protein as a model target, the HTS of the libraries obtained in three selection cycles revealed three to eight times more affine aptamers in comparison to the Sanger sequencing 98 clones from the final library [19]. Thus, HTS implementation in SELEX was proved to provide more successful aptamer candidate identification compared to conventional Sanger sequencing.

The large number of sequence reads derived from the HTS-assisted SELEX experiment is not the only key to success for the correct identification of aptamer sequences. As discussed above, several main strategies for candidate aptamer identification can be used in HTS data analysis. The sequences can be selected based on the maximum abundance criteria [53,54], scoring of the cycle-to-cycle enrichment [19], cluster analysis [15], and sequence and structural motif accumulation [23,24].

The initial idea of the SELEX experiment presumes that the binding oligonucleotides are retained by the target and amplified in PCR, thus the binders become substantially enriched in the selected pool. In practice, the prevalent sequences can originate from PCR bias [19,24,25], non-uniform sequence distribution in the initial nucleic acid library [35], energy-driven selection of particular structures [40,55], and non-specific background binding [35]. The realization and degree of influence of all these factors is rather case-specific and also tends to increase with the selection cycle number [24].

Thus far, many aptamers have been successfully recognized using the maximum abundance as the main criteria for selecting candidate binders; this strategy is sometimes used with HTS-assisted selection [53,54,56]. However, there is always a chance that the best binders are underrepresented [57], and here, the power of the HTS experiment can be realized to a greater extent. For example, in a capture-SELEX experiment against tobramycin, the sequences obtained using HTS from the terminal cycle were clustered, and the representatives of each cluster were selected as the aptamer candidates [17]. All displayed target binding, and no correlation with the abundance was detected [17]. Cycle-to-cycle relative scoring of the aptamer abundance or enrichment ratio proved to be even more efficient for aptamer identification [19].

In developing the HTS-assisted microfluidic selection platform against the PDGF-BB protein as a model target, Cho et al. experimentally evaluated the candidates identified using abundance and enrichment-fold criteria. The most abundant sequences demonstrated significantly lower affinity in comparison to the ones identified by the enrichment fold score [19]. The benefits of the motif search approach were demonstrated by P. Jiang et al. [24]. Using the HTS dataset obtained from selection against human embryonic stem cells, the 6-mer motif search was performed. The motif abundance was scored, and the sequences in the dataset were ranged based on this scoring, depending on bearing the most popular motifs. The predictive power of this aptamer ranging appeared to be very acute, while the top abundant sequence did not display target binding [24]. All these results demonstrate the advantages of HTS to fit the main goal of SELEX experiments.

### 5.2. Decreasing the Number of Selection Cycles

The second major benefit of HTS implementation with SELEX is the possibility of identifying the binders while performing fewer SELEX cycles. Almost all reports on HTS-assisted SELEX experiments mention the early emergence of binding sequences. For example, streptavidine aptamers could already be identified at the third round of selection, while the selection was performed in 10 cycles [31]. APTANI Suite was able to identify the known aptamer specific for murine IL4Ra protein [58] in the HTS dataset obtained after the first selection cycle [23]. The accumulation of binders often correlates with the appearance of minimal library enrichment that can be indicated with the pool diversity loss or the emergence of pool affinity toward the target [17,51].

The emergence of target-binding aptamers at early selection cycles is often demonstrated retrospectively, after the selection has already been performed with excessive cycles. Some efforts have been made to develop approaches to introduce selections with less cycles. Successful isolations of aptamers within a single selection cycle have been reported, and, in this case, HTS becomes an indispensable selection instrument. For example, a MEME-assisted motif search using HTS derived data allowed for the identification of similar motifs both within one and five selection cycles aimed at developing a bivalent thrombin aptamer [59].

S. Hoon et al. identified effective thrombin binding aptamers in one selection round using the specially developed algorithm of HTS data analysis based on K-mer scoring [18]. Capillary electrophoresis (CE)-based non-SELEX isolation of DNA aptamers against tau Proteins also employed HTS and was performed with only three selection rounds, where HTS was the essential instrument for the identification of candidate sequences [60]. The selection protocol called RAPID SELEX, based on a combination of non-SELEX and SELEX steps, also relies on HTS data analysis [61].

### 5.3. Validation of the Starting SELEX Libraries

The quality of the starting nucleic acid contributes to the success of the aptamer selection process [3,62]. HTS offers the opportunity for extensive analysis of the random libraries. Single HTS experiments are typically unable to cover the diversity of the initial library used in SELEX because it contains more unique species then can be covered within a sequencing chip; however, if the library is biased, this can be detected with deep sequencing [63]. The nucleotide distribution during the chemical synthesis of randomized regions depends on the molar ratio of A:C:G:T phosphoramidites [64] and even the method of mixing (by hand or automated) [65] and supplier choice [63]. The length of the random region and desired nucleotide distribution can be perfectly confirmed using HTS [63,65].

### 5.4. Study of Sequence and Nucleotide Bias in the Selection Process

SELEX is a complex process requiring many experimental steps and, due to this, is known to have many pitfalls [3]. HTS can serve as a very efficient instrument for the identification and assessment of the factors influencing the efficiency of aptamer selection.

B. Zimmermann et al. performed a neutral SELEX–genomic SELEX experiment omitting the target binding step [55]. All other procedures were kept the same as for targeted selection with genomic RNA SELEX against the *E. coli* regulator protein Hfq. In both targeted and non-targeted selections, the same RNA library derived from *E. coli* genomic DNA was used. Library pools obtained from ten cycles of neutral SELEX were sequenced using the 454 pyrosequencing platform, and the results were analyzed and compared to the HTS data obtained from the ninth cycle of targeted selection. Analysis of sequencing results allowed for the derivation of important information on how non-targeted selection pressure imposes evolution factors on the library. With an increasing selection cycle number, the average length of selected sequences decreased.

The selected sequences became more and more distant from the initial genomic sequence, which indicates that the mutations accumulated during selection. The structural stability of the selected library species tended to decrease under the selection process. At the same time, the library diversity in the neutral SELEX experiment was strongly preserved in comparison with the targeted selection. In the target-selected pool, fewer sequence clusters were identified, and these clusters contained more representatives. The pools obtained from targeted and non-targeted selections differed strongly in their nucleotide and dinucleotide composition.

Under neutral SELEX, a loss of G, a strong increase in A, and a slight increase in the U content were detected. With respect to the dinucleotides, the AA, AU, and GU content increased, while the CG, GC, and GG content decreased. These changes can be further attributed to the selectivity of the selection steps rather than to mutations. The results clearly indicate that the selection steps themselves influence the selection process. Neutral SELEX also resulted in the enrichment of certain sequences. The authors suggested deducting these representatives from the targeted selection to refine the results.

A similar HTS experiment compared non-targeted and targeted selections against different mammary carcinoma cell lines using an RNA library with a 51 nt length of the random region [66]. The changes in the nucleotide and dinucleotide distribution detected in this experiment also indicated the presence of bias in non-targeted selection, though the favor was exhibited for the accumulation of pyrimidines, while the A content decreased. This difference can be attributed to the nature of the initial library and influence of primer binding sites [66]. Both experiments indicated the non-targeted propagation of certain sequences.

Another example of HTS study of non-targeted selection was performed by P. Jiang et al. [24]. The results clearly demonstrated that the most abundant sequences identified in SELEX could be induced by PCR bias [24]. The degree of PCR bias can be stochastic and occurs accidentally. Consistent with the results derived from neutral SELEX [55], this research also provides evidence that target-induced enrichment results in changes in the motif composition, and this serves as a much more efficient predictor of target affinity of the selected aptamer candidate.

The HTS-based analysis of CE-SELEX against rhVEGF165 revealed the emergence and accumulation of short sequences from PCR amplification [25]. The formation of this by-product occurred both in bulk PCR during the SELEX procedure and in emulsion PCR during the preparation of sequencing libraries for 454 pyrosequencing. Based on the sequencing results, the authors attributed the appearance of these sequences to the failure of GC-rich sequence accumulation. The accumulation of by-products can be a reason for the drop in the pool affinity observed with the increasing selection cycle number [25].

The HTS-assisted evaluation of the difference between the in-solution standard PCR (openPCR, oPCR) and emulsion PCR (ePCR) amplification of SELEX-derived nucleic acid pools also revealed the loss of GC-rich fragments during oPCR-based selections [67]. ePCR resulted in the preservation of the library diversity. oPCR failed to amplify approximately 67% of the sequences with a strong tendency to lose GC-rich ones [67]. Similar results were reported in the extensive study of the PCR amplification process of randomized RNA [63] and DNA [66] libraries using HTS. Interestingly, the selection of DNA aptamers for ovarian cancer biomarker CA125 resulted in the isolation of four sequences that were characterized as aptamer candidates [65]. Two of them with 85% AT content (and not containing G at all) did not exhibit target binding. Two more candidates with normal CG content turned out to be target-specific aptamers [65].

The analysis of HTS data obtained through the selection of the nuclease-resistant RNA aptamers against the murine IL-10 receptor revealed that high-affinity binders can be lost during the enrichment due to being displaced with lower affinity aptamers, possibly due to the unfavorableness of the high affinity aptamers in PCR or a competing enrichment of other aptamers binding to another apatope of the target protein [52]. In the same experiment, the emergence of novel sequences arising from single-point mutations produced by the enzymes during the selection steps was detected. Some of these mutations were shown to accumulate, contributing to the overall pool evolution [52]. The study of the mutational landscape was further adopted to develop AptaMut, a method that serves to identify mutations that improve the binding affinity of the aptamers [22].

Taken together, the results of the selection bias studies derived from HTS experiments provide a basis to avoid using the abundancy of the sequence as the main predictor for candidate aptamer identification and to put efforts toward improving the partition efficiency and minimizing the selection cycles.

### 5.5. Comparison and Validation of SELEX Strategies

The design of the aptamer selection experiment is crucial for successful aptamer isolation. The choice of SELEX method depends strongly on the target type, its intended application, and the available equipment. The appropriate aptamer isolation strategy can result in the minimization of selection cycles and in the isolation of more affine aptamers [3]. HTS can serve as an instrument for the comparison of selection strategies and for the validation of newly developed SELEX protocols.

To compare the efficiency of differently designed selections and the rate of differently designed SELEX experiments, the rate of library diversity loss is usually analyzed it is believed to indicate the enrichment process. The number of unique reads in the sequenced pool was used to decide on the most effective strategy in the selection of DNA aptamers against testosterone [67]. The HTS results revealed that adding two selection cycles resulted in the strong enrichment of target-binding species [67].

The same approach for interpreting the HTS data was used to prove the efficiency of sol-gel SELEX for the isolation of DNA aptamers to xanthine [68]. No library enrichment was indicated after 14 cycles of bead-based selection against the same target, while using a sol-gel immobilization free strategy enabled the strong enrichment of the aptamer sequence in just seven rounds [68]. Microbead-assisted capillary electrophoresis (MACE) was shown to be superior over standard CE in SELEX against thrombin based on 43% enrichment of the best binder after three selection rounds of MACE SELEX in comparison to 0.16% in CE SELEX [69].

Another approach was described for the validation of the novel selection strategies. To prove the efficiency of selection, thrombin was used as a model target, and the starting library was doped with the known thrombin-binding aptamer (TBA) sequence. Under the experiment, the HTS data were analyzed for the enrichment rate of this known sequence. Using this approach, capillary transient isotachophoresis (ctITP)-based nonequilibrium capillary electrophoresis of equilibrium mixtures (NECEEM) was shown to enrich TBA from 0.4% in the starting library to 15% after the single selection cycle, where this result was reproduced with three different selections [11].

### 5.6. Assisting Novel SELEX Strategies

SELEX is being extensively developed to fit new goals and new modifications of the protocol appear each year. As HTS becomes increasingly popular, it often an indispensable tool for novel aptamer isolation strategies.

Genomic SELEX utilizes genome-derived nucleic acid (both DNA and RNA) libraries for aptamer isolation [7]. Such libraries are somewhat constrained in diversity, but initially have an increased chance of finding the sequences interacting with the DNA and RNA binding properties. Genomic SELEX is an efficient tool for the discovery of fragments of the genome or transcriptome interacting with the target of interest, which can be a regulatory protein or other ligand known to interact with cellular nucleic acids [7]. Binding sequences identified with SELEX will not represent the whole functional domain of the genome or transcriptome, but only a specific short “aptamer” sequence binding to the target [7,70].

The recognition of the domain demands additional efforts. There is a chance that the natural regulating target interacts with more than one or several binding sites. Thus far, in genomic SELEX, HTS is a favorable instrument. The large number of reads is helpful in identifying multiple binding sites; and the possibility of aligning the sequencing results to the initial genome can serve in the correct identification of functional domains [7]. In addition, the initial genome-derived libraries tend to contain overrepresented sequences, and this can easily be taken into account with the HTS of the initial pool, which further allows normalization of the reads obtained for the SELEX-derived pools [7].

The potential of HTS with genomic SELEX was demonstrated by C. Lorentz et al. [71] in the selection against the *E. coli* global regulator protein Hfq using the RNA library transcribed from the *E. coli* genome. The HTS-derived data were used to identify the binding motif using MEME and to map the enriched sequences to the *E. coli* genome. The abundance of enriched fragments was normalized to the data obtained from neutral, non-targeted SELEX experiments performed using the same conditions [55].

Based on the results of this HTS data analysis, the Hfq-binding RNA motif was recognized and then experimentally proven to bind its target with a nanomolar dissociating constant. Novel Hfq targets were identified, and the mechanism of Hfq regulation of the expression of a large number of genes via interaction with cis-antisense RNAs was suggested [71]. The same approach was used to determine the binding sequence and regulation mechanism of the PapX *E. coli* protein [72]. A special software called transcription factor analysis using SELEX with high-throughput sequencing (TFAST) was developed to assist in HTS-based genomic SELEX experiments [73].

The ability to sequence a tremendous number of sequences provided by HTS opens the horizons for the extensive enlargement of targets for parallel selections. As an example, the procedure for the identification of binding sites for hundreds of DNA binding domains of transcription factors of *Ciona robusta* using genomic SELEX was described [74]. With the aid of HTS, authors were able to recognize 139 recognition sequences, which is an extensive contribution to the study of the gene regulation process. Though demonstrated using genomic SELEX, the idea of massive parallelization of targets can be exploited for other selections.

Most SELEX approaches are generally based on sequence abundance scoring, and due to this, rare binders can be lost during selection even if the scoring results are corrected with normalization using non-targeted SELEX [55]. To overcome this limitation, a novel experiment design called Apta-Seq was developed by M. M. Abdelsayed et al. [75]. The idea of Apta-Seq lies in chemical mapping of these unstructured (unpaired) RNA nucleotides using 2′-hydroxyl acylation of RNA. If an RNA aptamer changes its structure upon target binding, the positions ready for chemical modification can change in a target-concentration-dependent manner. In terms of the experimental procedure, the selected RNA pool is divided into equal portions and incubated with different concentrations of the target and then submitted to a chemical modification reaction.

The positions (modifications of which were dependent on the target concentration) can be further identified, and this serves in the recognition of the exact sequence responsible for target binding. The identification of modified positions is performed using HTS. For this purpose, the reverse transcription (RT) of the modified pools is performed, and the modified positions serve as stopping points of RT. The reverse transcribed pools are then barcoded and sequenced using HTS. The analysis of sequencing data is intended to find the stop positions and reveal their dependence on the target concentration. This procedure is especially relevant to the identification of structure-switching RNA aptamers and can be generalized to any RNA-based in vitro selections [75]. Apta-Seq enables immobilization-free selections and provides data on the aptamer affinity and its structural features, and HTS is the essential instrument in utilizing this approach.

### 5.7. Deeper Analysis of Aptamer Structure and Function

The data obtained from HTS can serve for post-SELEX optimization of the aptamer structure. Shortening of the aptamer sequence is a procedure that can be performed with two main strategies [52]. The first and less effective one is to trim a few nucleotides from both ends and then evaluate the aptamer binding to the target. The second and more efficient way is to analyze the aptamer folding, divide it into a few blocks, and study the target binding of these structure fragments. The computational folding of RNA and DNA is typically based on the principle of minimal free energy. In some cases, multiple possible structures with nearly the same energy are predicted, and it is difficult to understand which of them is realized.

For the aptamer binding to the murine interleukin-10 receptor isolated using HTS-based SELEX, three probable structures were predicted using RNA folding prediction software, and the structures were referred to as alpha, beta, and gamma [52]. The analysis of the HTS data revealed the appearance of single-point mutations during the selection process, and some of these mutations were enriched due to their target affinity. Under the hypothesis that the survived mutations should not destabilize the aptamer structure, the authors analyzed the influence of mutation to the aptamer structures. The gamma structure was shown to be less affected by all the mutations and thus predicted to be realized in nature under the selection conditions. Using this structure as a rationale for truncation, the authors succeeded in shortening the aptamer structure from 80 to 48 nt [52].

An effective and intelligent analysis of HTS data aimed to maximize the information on aptamer structure and function was performed by D. M. Dupont [76]. In the previous research, two effective aptamers for the serpin plasminogen activator inhibitor-1 (PAI-1) called paionap-5 and paionap-40 were identified with Sanger sequencing after five selection cycles, and both the amino acid residues of PAI-1 essential for aptamer binding and the binding structural motifs of the aptamers were identified [77]. To extend this work, the selection was continued with one more cycle in a branched mode against the range of mutant forms of PAI-1 within the position identified as contributing to the target binding [76].

The pools before and after the branched selection were sequenced with the Illumina platform. Respecting the idea that the abundance of the aptamer sequence before and after the selection cycle reflects its affinity to the target, the enrichment of two known aptamers after selection against the target mutants was analyzed. The results of this analysis were consistent with the experimentally derived data; the enrichment ratio of the aptamers was less efficient toward the mutants in comparison to the enrichment with the wild-type PAI-1. Each aptamer was characterized with the pattern of enrichment, which was the difference between the enrichment toward each of the mutants and wt-target.

The HTS dataset was scanned to find other sequences with the same pattern of enrichment as the known aptamers. This search identified 97 new sequences with the same enrichment patterns called paionap-5, and 82 species with an enrichment pattern similar to the paionap-40 aptamer. The MEME motif search derived the common motifs in both aptamer groups, and these motifs were the same as previously identified using biochemical approaches [77]. The structures of both aptamer groups were predicted. For paionap-5-like motifs, several structural families were identified in which the binding motif folded into a loop structure. For the paionap-40 aptamer family, the binding motif formed the only three-way junction structure. Based on this structural analysis, the shortened versions of the binding motifs were created, which retained the target-binding properties of the initial aptamers.

In addition, HTS of the pools before and after the branched selection was used to identify the two residues in PAI-1 that were hot-spots of oligonucleotide enrichment as many of the sequences enriched with wt-PAI-1 were not enriched with the two mutant forms. Finally, one of the selection branches was performed against the complex of PAI-1 with vitronectin, and the differential analysis of the enrichment in two pools (against the wt-target and PAI-1-vitronectin complex) enabled the identification of novel aptamers binding to the PAI-1-vitronectin complex, while paionap-5 and paionap-40 did not exhibit such binding. Therefore, the extensive analysis of HTS data was exploited to investigate the aptamer affinity and specificity and to reveal its structural and functional characteristics [76].

The prediction of aptamer specificity based on the evaluation of the difference in enrichment toward different targets was also successively used for the development of several aptamers. This approach is based on the hypothesis that aptamer preferential abundance is related to the specificity. DNA aptamers to the steroids estradiol, progesterone, and testosterone were selected in one-pot SELEX and then identified based on HTS analysis of the pools obtained from the differential selection step [78]. The revealed aptamers displayed the desired specificity [78]. Comparison of the enrichment ratios of the sequences obtained for positive and negative selection cycles enabled the identification of the DNA aptamer binding specifically to the desired target in cell-SELEX [79,80]. The divergence of the selection at the terminal step to the target and negative selection with the following HTS helped to reveal the unspecific binders in the SELEX of RNA aptamers against the human interleukin (IL)-10 and human 4-1BB receptors [81].

Thus, the intensification of HTS data analysis can result in obtaining further information regarding the structure and function of aptamers.

## 6. Conclusions

The implementation of HTS with SELEX provides an impressive potential for aptamer discovery, characterization, and post-SELEX optimization. HTS-SELEX serves in the precise prediction of target binders within fewer selection cycles. The extensive analysis of HTS data facilitates tuning the affinity and specificity of aptamers and assists in a deeper understanding of the aptamer structure and interaction with the target. The SELEX process itself and satellite methods can be investigated using HTS. Due to the expansion in HTS, novel HTS-SELEX methods appeared, which cannot be realized with conventional sequencing. The flip side of HTS employment is its relatively high cost and the requirement to proceed with and analyze the huge amount of data.

The expenses associated with performing HTS experiments decrease year by year due to the constant developments in this field and growing competition between the manufacturers of sequencing devices. The future potential for HTS cost decreases resides in the adaptation of other sequencing methods with a lower price, like nanopore sequencing. The cost per single sequence of a library species in an HTS experiment is lower than in Sanger sequencing. To overcome a second drawback—the need for analyzing tremendous datasets—different bioinformatics tools have been developed.

Various instruments for users with different computer skills and levels of bioinformatics expertise are available. These instruments serve in aptamer sequence identification and rely on several different approaches for this goal. All of these approaches have been shown to provide successive results in the aptamer selection process; however, there is a need for the systematic comparison of their predictive performance. For this purpose, de novo comparative experiments with the same range of targets are demanded, while, at the moment, the validation of each method/program is typically performed with a retrospective analysis of the datasets obtained in the preceding aptamer isolations with a priori knowledge of the aptamer sequences.

The feature set of HTS-SELEX specialized software in the future can be expanded to realize the power of HTS experiments to a greater extent. For instance, the specific analysis of HTS-SELEX results aiming to investigate aptamer structure and the function relationship and aptamer specificity is performed with the hand data operation [52,76,78,79,81] or with the adaptation of non-specialized tools [80]. The accounting of negative and non-targeted selections required for aptamer specificity control as well as the analysis of the binding patterns of the sequences can be integrated in software tools. The analysis of the aptamer structure appears to be too complicated and too creative a process to be automatized at this time.

One of the issues to be addressed in structure motif searches is the difference between DNA and RNA folding, with respect to the fact that all software packages performing the search of structural motifs are based on RNA-specific algorithms. None of the available software tools offer the ability for masking or deletion in the internal region of the sequence, which can be helpful for some datasets such as those derived from capture-SELEX experiments or aptamer selections using libraries with pre-defined structures.

To make HTS more widespread in aptamer selection, the availability and affordability of software instruments for researchers without advanced computer skills and bioinformatics expertise must be enhanced. The developers of software tools can put effort toward providing specific data analysis protocols, like the one published by Thiel et al. for the Galaxy resource [28]. Further integration and improvement of the graphical user interface to the specialized software packages is a trend in HTS-SELEX bioinformatics to fit the goal of software accessibility. In addition to the GUI, easy and cross-platform installation is required, which can be alternatively afforded with Web-based resources as realized by Galaxy or MEME.

With increased capabilities for aptamer sequence identification, the experimental characterization of aptamers becomes a bottleneck of SELEX experiments [82,83]. Among the thousands of sequenced species, hundreds become strongly enriched, and dozens of clusters can be identified among them. Not all of them exhibit target binding due to a variety of reasons including non-specific or biased enrichment or cooperative target binding with other oligonucleotides. Therefore, the number of aptamer candidates for experimental evaluation in HTS-SELEX experiments becomes rather high. When using different aptamer recognition strategies in a single experiment, this number can be multiplied. Currently, methods for parallel and fast aptamer characterization are required to assist HTS-SELEX. Certain high-throughput platforms for aptamer evaluation have been developed [84,85]; however, novel methods are still required.

With respect to the significant propagation of HTS applications in SELEX and the speed of the development of specialized software tools during the recent years, we believe that HTS will soon become a routine instrument in aptamer isolation technology and contribute strongly to both the speed of aptamer discovery and to the growing scope of knowledge regarding aptamer structure and function.

## Figures and Tables

**Table 1:** Features of the software tools developed or adapted for high-throughput sequencing data analysis in systematic evolution of ligands by exponential enrichment.

Software	Analysis Stages	Platforms	GUI	Source
Galaxy [28,29]	Data preprocessing	Web/Linux/Mac OS	Yes	https://usegalaxy.org/
	Enrichment counting			
FastAptamer [32]	Enrichment counting	Linux/Mac OS/Windows/Galaxy	No	https://github.com/FASTAptamer/FASTAptamer
	Sequence based clustering			
	Searching known motifs			
PATTERNITY-seq [33]	Sequence based clustering	Linux/Mac OS/Windows	No	https://github.com/AptaFred/EGE_tree
MEME/GLAM [37]	Sequence motif searching	Linux/Mac OS/Web	Yes (Web)	http://meme-suite.org
MPBind [24]	Data preprocessing	Linux/Mac OS	No	https://morgridge.org/research/regenerative-biology/software-resources/mpbind/
	Sequence motif searching			
Aptamotif [40]	Structure motif searching	Linux/Mac OS	No	By request
APTANI [23]	Enrichment counting	Linux/Mac OS	No	http://aptani.unimore.it/
	Structure motif searching			
APTANI^2^ [42]	Enrichment counting	Linux/Mac OS	Yes	http://aptani.unimore.it/
	Structure motif searching			
AptCompare [43]	Data preprocessing	Linux/Mac OS/Windows	Yes	https://bitbucket.org/shiehk/aptcompare/src/master/
	Enrichment counting			
	Sequence based clustering			
	Sequence motif searching			
	Structure motif searching			
COMPAS [45]	Data preprocessing	Unknown	Yes	Unknown
	Enrichment counting			
	Sequence based clustering			
	Structure motif searching			
RaptRanker [46]	Data preprocessing	Linux/Mac OS	No	https://github.com/hmdlab/RaptRanker
	Enrichment counting			
	Structure motif searching			
AptaSUITE [48]	Data preprocessing	Linux/Mac OS/Windows	Yes	https://github.com/drivenbyentropy/aptasuite
	Enrichment counting			
	Sequence based clustering			
	Structure motif searching			
	SELEX simulation			

## Abbreviations

HTS	High-throughput sequencing
SELEX	Systematic Evolution of Ligands by EXponential enrichment
ISFET	Ion-sensitive field effect transistor
NGS	Next generation sequencing
GUI	Graphical user interface
IT	Information technology
CE	Capillary electrophoresis
TBA	Thrombin-binding aptamer (TBA)
ctITP	Capillary transient isotachophoresis (ctITP)
MACE	Microbead-assisted capillary electrophoresis (MACE)
PAI-1	Plasminogen activator inhibitor-1 (PAI-1)
